# St. Bartholomew's Hospital Action

**Published:** 1916-02-19

**Authors:** 


### HOSPITAL ECONOMICS IN WAR-TIME.

### St. Bartholomew's Hospital Action.

Now more than ever is it necessary for the hospital administrator carefully to examine every part of the machinery of distribution of all classes of goods used in the daily work of the institution. It was with this axiom in mind that the treasurer and almoners of St. Bartholomew's Hospital issued, at the close of last year, to the heads of all departments, a list showing prices then paid for provisions, drugs, and most other articles in daily use, as compared with rates before the war. This list was accompanied by a statement dwelling upon the importance of the exercise of strict economy wherever such could be effected without prejudicing the welfare of the patients or the general efficiency of the hospital.

Buying at the present time is a difficult art. One has to search for markets in which to get reasonable terms; the pre-war conditions under which sellers brought their wares to offer to the institution are temporarily suspended. Buying, however, is in the hands of the few; the secretary, the steward, the matron, and the pharmacist are the people chiefly concerned in the operation. The use of goods is dependent upon the discretion of a far larger number of persons, from the heads of the departments down to scrubbers, ward-maids, porters, and stokers. Practically every article in daily use has increased enormously in price, in some instances several hundreds per cent., and it is essential that these very valuable stores shall be employed with the greatest care and to the best advantage. Nothing brings home to the individual a fuller idea of the value of commodities than an exact knowledge of their cost. The action, therefore, of the authorities of the great Metropolitan hospital referred to is one which might very well be emulated in many directions.

### Before and After.

The prices before us are described as "Prices before the war" and "Present prices," but they obviously refer in some instances to contract prices, and in others to the cost of articles that were the subject of forward purchases. For instance, at the beginning of December last it was not possible to buy sodii bromid. at 7s. per pound. However, as the prices are of general interest, we print in the next column extracts from the two-page foolscap list distributed in St. Bartholomew's Hospital in December.

### Checks on Expenditure.

The science of distribution has been carefully studied at many institutions. In "The Uniform System of Accounts of Audits and Tenders for Hospitals and Institutions," a new edition of which will shortly be published, is to be found a careful explanation of the system in vogue at a large Scottish infirmary, under which it is possible to bring directly to the notice of a physician and surgeon the exact cost of maintenance, etc., of the patients under their care as compared with the patients of other members of the honorary medical staff. In many hospitals the same economical method has been adopted, and in some cases, instead of being applied to groups of patients, the calculations are made in respect of wards.

Amongst the main spending departments the chief at the present time in many institutions is the dispensary, and where there is a large out-patient department an unusually watchful eye has to be kept on issues. Here, again, a system is necessary under which the out-patient physicians and surgeons, casualty officers and others shall be informed of the cost of the drugs dispensed for and the dressings used in each room, or as to the average calculation of cost in respect of the patients of each department.

Some indication of how heavily the prices of drugs is affecting the cost of treatment of the humbler sick members of the community finds expression in the fact that many panel patients who for a time ceased attending the voluntary hospitals are now coming back again, their panel physicians having found that they cannot consistently with the welfare of the patients prescribe for them within the restricted financial limits of the State scheme.

*Table showing Present and Pre-war Prices of Provisions, Drugs, etc.*

| | Prices before the War | | Present Prices | |
|---|---|---|---|---|
| **Provisions:** | s. | d. | s. | d. |
| Beef ... ... ... | 0 | 5 per lb. | 0 | 9 per lb. |
| Mutton ... ... | 0 | 5 ,, | 0 | 8 ,, |
| Bread ... ... ... | 10 | 6 per cwt. | 16 | 0 per cwt. |
| Butter ... ... | 1 | 0½ per lb. | 1 | 5½ per lb. |
| Bacon ... ... ... | 0 | 8½ ,, | 1 | 0½ ,, |
| Milk ... ... ... | 0 | 9½ per gallon | 1 | 0 per gall. |
| Sugar, Loaf ... ... | 0 | 2 per lb. | 0 | 4½ per lb. |
| **Fuel:** | | | | |
| Coal, House ... ... | 19 | 6 per ton | 28 | 11 per ton |
| ,, Steam ... ... | 21 | 11 ,, | 29 | 0 ,, |
| **Drugs and Dressings:** | | | | |
| Acid. Aceto-Salicyl ... | 1 | 8¾ per lb. | 45 | 0 per lb. |
| Acetone ... ... | 0 | 6 ,, | 2 | 0 ,, |
| Atropine Sulph. ... | 11 | 3 per oz. | 85 | 0 per oz. |
| Sodii Salicylas ... | 1 | 2½ per lb. | 20 | 0 per lb. |
| Sodii Bromid. ... | 1 | 10 ,, | 7 | 0 ,, |
| Phenacetin ... ... | 3 | 0 ,, | 58 | 0 ,, |
| Zinc Oxid. ... ... | 29 | 6 per cwt. | 90 | 0 per cwt. |
| Plain Lint ... ... | 1 | 0 per lb. | 1 | 6 per lb. |
| **Glass:** | | | | |
| Excreta Pans ... | 2 | 0 each | 4 | 0 each |
| Specimen Glasses ... | 3 | 0 per doz. | 7 | 0 per doz. |
| Graduated Glass Measures ... ... | 0 | 6 each | 1 | 0 each |

